# Report of the Committee on Army Legislation1Read before the American Veterinary Medical Association, New York, September, 1899.

**Published:** 1899-09

**Authors:** D. E. Salmon

**Affiliations:** Chairman


### REPORT OF COMMITTEE ON ARMY LEGISLATION.[1]

Mr. President: Your Committee on Army Legislation would respectfully report that in the early part of the last session of Congress the desirability of legislation improving the status of the army veterinarian was brought to the attention of the Committee on Military Affairs of both the House and the Senate. The importance of securing such action by Congress in the bill for the reorganization of the Army was fully appreciated, and a circular letter was addressed to the members of our profession in the United States, asking that their influence be exerted to aid in securing this result. By the courtesy of Dr. W. Horace Hoskins, one of the members of the committee, a very complete list of veterinarians was placed at the disposal of the committee for this purpose. It was possible in this way to reach something like five thousand gentleman, who from their profession and experience would naturally be interested in and appreciate the value of a properly equipped and organized veterinary service in the army.

The response to this appeal to our profession was extremely gratifying. Not only did a large number of members write to their Senators and Representatives explaining the necessity to good service of the legislation desired, but they generously assisted the committee with funds to pay for printing, postage, and other expenses, so that the treasury of this Association was not drawn upon.

The chairmen of both committees of Congress on Military Affairs were favorable to legislation giving increased compensation and rank, and it is believed that the clause in the bill as reported, providing the veterinarians with the rank, pay, and allowances of a second lieutenant of cavalry with each cavalry regiment, would have passed without opposition had it not been for the unfortunate defeat of the entire bill by a compromise measure. The compromise bill originated in the Senate, and the chairman of your committee was given a hearing before the sub-committee which had the measure in charge. It was late in the session, time was very limited, many interests were to be heard, and while the profession did not secure all that it desired, your committee is of the opinion that there is reason for encouragement rather than despair.

The bill as passed provides for two veterinarians with each regiment of cavalry, one to have the pay and allowances of a second lieutenant of cavalry, the other to have the pay of $75 per month and the allowances of a sergeant-major. It is also provided that the veterinarian appointed to the first grade shall not be so appointed until he shall have passed an examination, to be

[1] Read before the American Veterinary Medical Association, New York, September, 1899.

prescribed by the Secretary of War, as to his physical, moral, and professional qualifications. There is a further provision that the veterinarians now in the service who do not pass such competitive examination shall be eligible to the positions of the second class under such rules as are now prescribed by the regulations.

It is to be sincerely regretted that the veterinarian of the first class is not given the rank as well as the pay and allowances of a second lieutenant of cavalry. A step has, however, been made in the right direction, the merit system is recognized by the inauguration of examinations, provision has been made for the retention of deserving men who have long and faithfully served in the army and who may not be able to pass the examination, and it is believed that there will be a disposition in the next Congress to do something more.

Your committee especially desires to express its appreciation of the encouragement and assistance which it received from the Hon. James Wilson, Secretary of Agriculture, and of the courteous treatment and sympathy extended by Hon. J. A. T. Hull, Chairman of the House Committee on Military Affairs. Many members of both houses of Congress have expressed their interest in the effort to secure an efficient veterinary organization for the army and their willingness to aid whenever the opportunity offered.

Your committee has endeavored in its work to show the importance of having an organized veterinary service which should extend to the artillery and Quartermaster's Department as well as to the cavalry. It has also tried to make plain the fact that a few veterinarians holding inferior positions in the cavalry regiments, necessarily scattered over the country, having no connection with each other and without means of concerted action, could not be expected to meet the more serious veterinary problems with which the War Department is often confronted. Such veterinarians in the past have reported that they could neither obtain proper medicines nor instruments; that they had no authority to cause the treatment which they prescribed to be carried into effect; that not being consulted in the purchase of horses, animals with contagious diseases were often introduced, and much damage was frequently caused before the contagion could be controlled.

The present veterinary service of the army is undoubtedly a disgrace to an enlightened and progressive country. It is a service which, to accomplish anything, must be able to carry its directions into effect, and yet it is without rank or authority; it is a service which requires instruments and supplies of a special character, and detailed instructions as to the manner of meeting the various emergencies which are liable to arise, and yet it is without a head; the veterinarian must endure all the hardships and face all the dangers of the service, and yet neither he nor his family have any prospects of a pension in case of disability or death in the service.

The result of this anomalous condition of affairs was very apparent in the course of our short war with Spain. The Government corrals became hot beds for the production and dissemination of glanders, and the efforts to check this disease were in some cases so crude that they might provoke a smile of derision on the countenance of our enemies, but could only bring a blush of shame and indignation to the face of a humane American citizen. At one place in Florida weeks of time were spent in testing the animals with mallein, and yet horses which showed unmistakable symptoms of glanders upon the most superficial examination were not separated from the healthy ones, and nose-bags were used indiscriminately. Injured and sick horses went without treatment because the veterinarians lacked medicines, instruments, instructions, and authority. There is little excuse for such a condition of affairs. While this is a rich country, and the loss of a few millions more or less on horses does not affect us seriously, it is, nevertheless, a humane country, and our people are not disposed to tolerate unnecessary suffering and cruelty to animals either by individuals or by the Government. The fact is the Government, through its various departments, should set an example of what is required in this civilized age in the way of intelligent and humane treatment of the animals which it controls. To accomplish this the army veterinary service needs to be reorganized; it needs a head. With the present service there can at the best be but such practice as can be conducted under unfavorable conditions by veterinarians who are willing to take positions with the pay of second lieutenants; but, with one experienced and really capable man at the head the entire service could be brought approximately to his level.

In conclusion, your committee mentions with much sadness the death of Dr. M. J. Treacy, who labored so long to improve the veterinary service in the army, who was for many years a member of this Association, and who died at his post of duty in Cuba from yellow fever. His career illustrates most vividly the dangers which the veterinarian in the army must face, and the ungenerous treatment which the country accords him. It is a pathetic incident in this connection that Dr. Treacy had studied hard and faithfully for the examination for the new position created in the recent legislation, and that, although he passed with the highest mark reached by any of the applicants, he did not live to learn of his success. On the very day that the examination papers were marked in Washington the news of his death came over the wires from Cuba. Let us, who are still living, see to it that his labors and sacrifices to secure intelligent and humane treatment for army horses, and at the same time to advance the standard of his profession, shall not be forgotten, and that the cause for which he died shall yet succeed.

D. E. Salmon,
Chairman.

